# Utilization of Telemedicine in Addressing Musculoskeletal Care Gap in Long-Term Care Patients

**DOI:** 10.5435/JAAOSGlobal-D-19-00128

**Published:** 2020-04-14

**Authors:** Olivia Cheng, Nok-Hin Law, Jessica Tulk, Michelle Hunter

**Affiliations:** From the Collingwood General and Marine Hospital (Dr. Cheng and Dr. Law); Rural Ontario Medical Program (Dr. Law and Ms. Hunter), Collingwood, Ontario, Canada; and Ontario Telemedicine Network, Toronto, Ontario, Canada (Ms. Tulk).

## Abstract

A notable proportion of patient transfers in Ontario are from long-term care facilities for consultation of musculoskeletal (MSK) issues. These transfers are costly for patients and the healthcare system. This study evaluated the utility of a telemedicine MSK (TeleMSK) service for long-term care patients requiring an orthopaedic consultation.

**Method:**

A cross-sectional study was used to assess TeleMSK from September 2018 to April 2019. Twenty-six long-term care facilities participated in this study, which included 32 long-term care patients assessed via TeleMSK and 27 telemedicine liaisons. The Telehealth Satisfaction Scale and the Telemedicine Usability Questionnaire (TUQ) surveys were used to evaluate the usefulness of the TeleMSK program.

**Results:**

Patients and families rated voice (64.3%) and visual (71.4%) quality of TeleMSK to be excellent as well as the length of consultation (92.9%). A total of 78.6% of participants rated explanations from physicians to be excellent and 92.9% of the participants rated the carefulness, skillfulness, respect, and sensitivity of the attending physician to be excellent 85.7%. Patients felt privacy and confidentiality was maintained and respected throughout the consultation. Most telemedicine liaisons agreed that TeleMSK improved accessibility and productivity of consultations, and 81.5% of the telemedicine liaisons strongly agreed that they would use TeleMSK again in the future.

**Conclusion:**

TeleMSK allowed for accessible, timely orthopaedic consultations without compromising the quality of patient care. Patients, families, and telemedicine liaisons rated their experience and the use of TeleMSK as excellent. Barrier to health care is an important issue in the long-term care population. TeleMSK is an excellent medium to close this gap.

A recent study on the sustainability of the healthcare system has shown that the ratio of healthcare spending compared with the Canadian gross domestic product has accelerated at an unsustainable rate between the years of 1998 and 2015.^1^ As the population of people older than 65 years of age increases, the projected expenditure on health care will increase markedly.^1,2,3,4^ Within the North Simcoe Muskoka Local Health Integration Network, the population of those older than 65 years of age represented 18% of the total population in 2015 and is projected to reach 24% by 2025.^4^ Owing to the current and anticipated increase in healthcare spending with the aging population, it is necessary to make changes that promote a cost-effective practice.^4,5,6,7^

Patient transfers to and from long-term care facilities were the highest of all interfacility transfers in Ontario (26.7%).^8,9^ Of these transfers, musculoskeletal conditions were the second most common reason for transfer with the first being due to circulatory issues.^10^ Transferring patients involves many risks including motor vehicle accidents, environmental hazards, and infection transmission. Furthermore, the unique demands of long-term care patient transfers are quite costly, averaging $450.00 per transfer. This cost varies depending on the distance traveled, if accompanying staff is required, and the wait time during the transfer. In addition, a family member often has to take time off work to accompany the patient for appointments.

Telemedicine musculoskeletal (TeleMSK) represents an initiative offering telemedicine orthopaedic consultations as an alternative to outpatient visits for individuals living in long-term care facilities.^11,12^ Telemedicine is advantageous in Canada because it has the potential to improve access to care for patients living in rural or remote areas and provide a cost-effective alternative to an “in-person” patient appointment.^11,13^ Specialists have reported that the use of telemedicine visits not only decreased the healthcare system's cost but also increased the number of patients seen while providing safe, effective, patient care.^12,13,14,15^ Orthopaedic surgeons have previously endorsed the usefulness of telemedicine for acute postoperative orthopaedic follow-up care, especially when in-person interaction is not feasible.^16^

Although there are many advantages to telemedicine, some concerns have been raised by healthcare professionals. Many orthopaedic surgeons have expressed concern about being unable to physically examine the patient and the safety of orthopaedic consults conducted via telemedicine. In addition, some orthopaedic surgeons think that patients and their families would prefer an in-person consultation rather than an encounter through the computer.

Previous prospective randomized control trials have shown no significant differences in patient satisfaction between telemedicine and in-person clinic visits. Telemedicine implementation in a rural orthopaedic setting to close the care gap in the long-term care population has not yet been examined. The purpose of this study is to evaluate the utility of telemedicine in providing musculoskeletal (MSK) care to long-term care patients. The unique challenges facing the long-term care patients include mental status, mobility, transportation cost, family, and care giver's ability to accompany and arranging for MSK care.

## Methods

A cross-sectional study was used to assess the orthopaedic telemedicine practice in the North Simcoe Muskoka Local Health Integration Network, which included 26 long-term care facilities. A total of 32 MSK consults were conducted by a single orthopaedic surgeon from September 2018 to April 2019. Family members were present for 10 of the consultations. After the consults, 32 patients and families as well as 27 telemedicine liaisons were invited to participate in the TeleMSK survey. For the purposes of this study, telemedicine liaisons were representatives from the Ontario Telehealth Network or a registered nurse working at a long-term care facility who acted as a facilitator for the patient and families during the consultation. Of the 32 consultations, 14 completed surveys were returned from the patients/family (% returned = 43.75%) and 27 completed surveys were returned from the liasons (% returned = 84.38%).

This study was approved by the local governing medical research ethics committee, and each of the participants was required to sign an informed consent form. When being invited to participate, potential participants were informed of the voluntary, anonymous nature of the study. They were informed that their participation or nonparticipation would not influence the medical care they received.

During the survey, two previously validated telemedicine satisfaction questionnaires were administered to the participants. After a TeleMSK consultation, patients and families completed the Telemedicine Satisfaction Scale (TeSS) questionnaire^17^ and the associated telemedicine liaison completed the Telemedicine Usability Questionnaire (TUQ).^18^ The questionnaires were collected and then returned to a local orthopaedic office where they were entered, analyzed, and securely stored in a locked-filing cabinet. Future documents and completed questionnaires will be destroyed once the study has been completed.

## Results

### Patient Satisfaction Questionnaire Results

**Figure 1:**
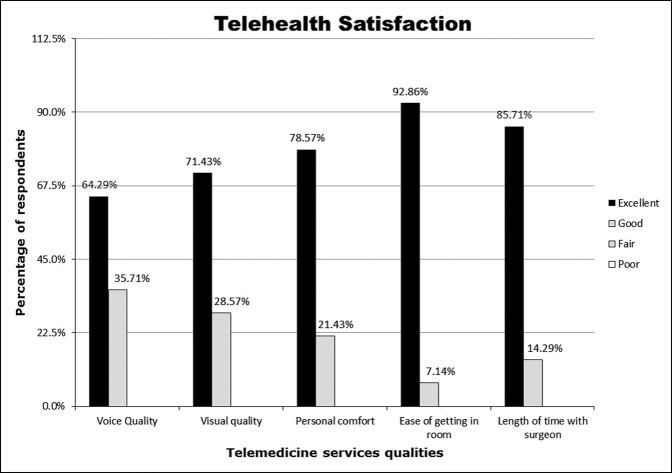
Chart showing the Telehealth Satisfaction survey bar graph (n = 14) regarding voice quality, verbal quality, personal comfort, ease of getting a room and length of time with surgeon.

Based on the results from the TeSS questionnaire, it is evident that most patients and families were satisfied with their experience using telemedicine as an alternative to in-office orthopaedic consultations (Figure 1). When patients and families were asked to evaluate the quality of the telemedicine encounter, 64.3% and 71.4% rated the voice and visual quality as excellent, respectively (Figure 1). Participants also rated the ease of accessing a room for the consultation (92.9%) and length of time spent with the surgeon (85.7%) to be excellent. Regarding the patient's comfort, 78.6% of the participants rated their comfort level with telemedicine as excellent, whereas the remaining 21.4% rated their comfort level as good.

**Figure 2:**
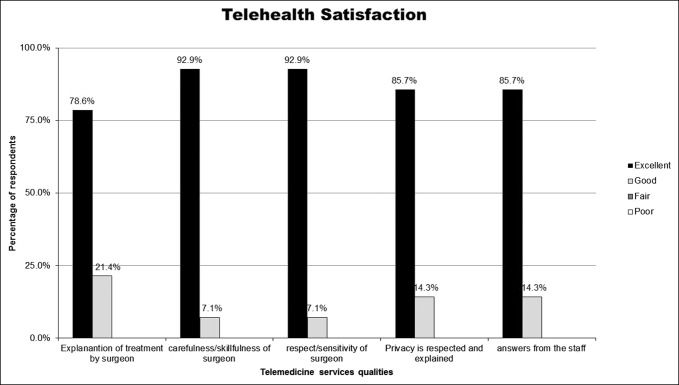
Chart showing the Telehealth Satisfaction survey bar graph (n = 14) with explanation, care, respect, privacy, and answers.

Patients and families were also asked to rate the quality of care that was provided by the attending orthopaedic physician and the liaisons that were facilitating the consultations over the telephone. As shown in Figure 2, most patients rated the quality of the telemedicine service as excellent and no patients rated the quality of care as fair or poor in any category. Most patients (92.9%) rated the explanation of treatment and the carefulness and skill of the attending physician or staff as excellent during their consultation. Moreover, most patients (85.7%) also felt privacy and confidentiality were maintained and respected while using TeleMSK. It is important to note that the results of the study indicate the quality of care was not compromised with the use of TeleMSK.

### Telemedicine Liaison Questionnaire Results

**Figure 3:**
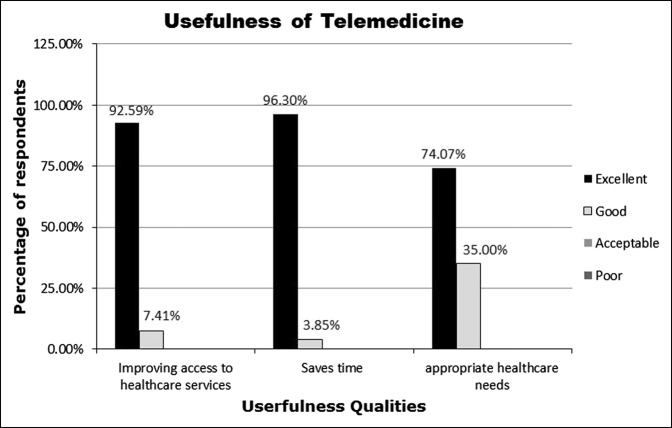
Chart showing the Telemedicine Usability Questionnaire (TUQ) bar graph (n = 27).

**Figure 4:**
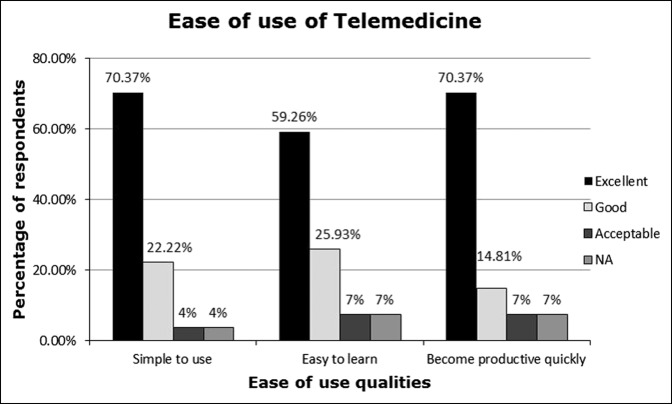
Chart showing the ease of use of telemedicine among telemedicine liaisons (n = 27). N/A = not available.

Overall, the TUQ survey results indicated that most telemedicine liaisons found TeleMSK improved access to healthcare services and saved time for patients and families (Figure 3). Among liaisons, 74.1% reported that TeleMSK addressed the patient's health care needs including accessibility, reducing unnecessary costs of transfer or travel, and improved efficiency in consultations. When conducting the consultations, the telemedicine liaisons noted that the program was simple to use and did not affect the delivery of quality care. Of the liaisons, 59.3% found it was easy to learn to use the telecommunication devices, whereas 22.2% noted it was moderately easy to learn (Figure 4). The majority (70.4%) found that the use of telemedicine helped improve productivity (Figure 4).

**Figure 5:**
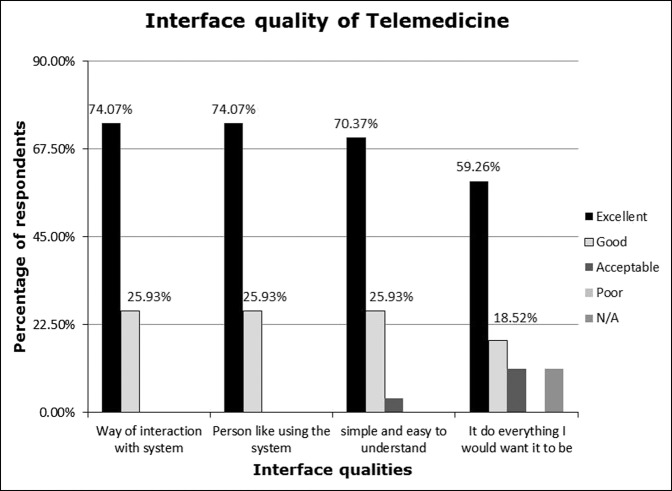
Chart showing the interface quality of telemedicine among the telemedicine liaisons (n = 27).

It is likely that productivity of consultations was partly improved because of the high quality of the telemedicine interface. Most liaisons (70.4%) noted that the interface was simple and easy to use and understand (Figure 4), However, just over half of liaisons (59.3%) rated the interface as excellent at being able to complete all functions the clinician wanted done during the consultation. Regarding reliability, 59.3% of the liaison rated the interface's reliability as excellent, whereas 18.5% rated it as good (Figure 5).

**Figure 6:**
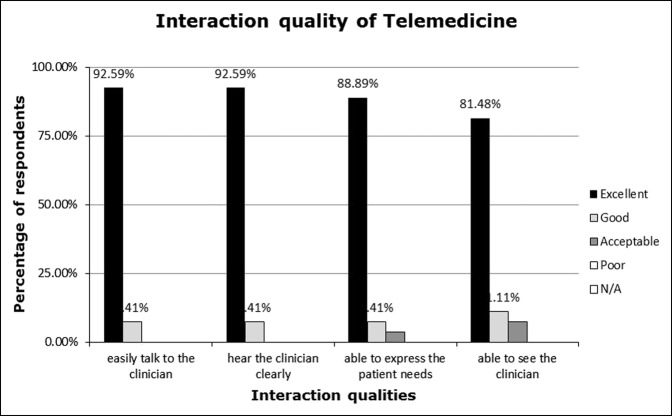
Chart showing the interaction quality of telemedicine among the telemedicine liaisons (n = 27).

Patients and telemedicine liaisons were also asked to rate the interaction quality throughout the consultation. The telemedicine liaisons noted that patients were able to easily talk to and hear the clinician and were able to express their needs clearly (Figure 6). In addition, 81.5% of the liaisons reported that the patients were able to see the clinicians clearly (Figure 6).

**Figure 7:**
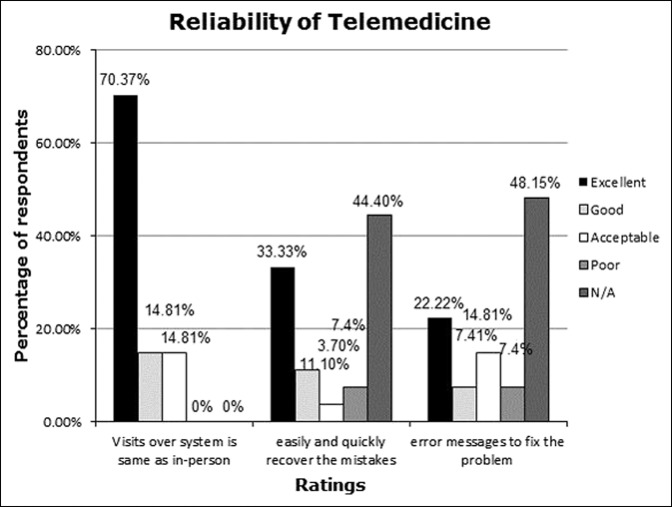
Chart showing the reliability of telemedicine among the telemedicine liaison (n = 27). N/A = not available.

**Figure 8:**
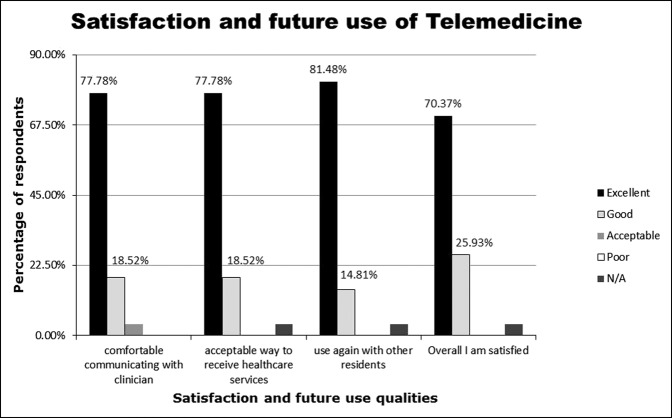
Chart showing the satisfaction and future use of telemedicine among the telemedicine liaison (n = 27). N/A = not available.

Finally, telemedicine liaisons were asked to evaluate the reliability of the TeleMSK program. Among liaisons, 70.4% rated the TeleMSK consultations as similar to an in-person consultation and 33.3% reported it was easy and quick to ameliorate any errors that arose (Figure 7). Overall, 81.5% of liaisons strongly agreed that they would use TeleMSK again in the future and 70.4% reported excellent satisfaction in using telemedicine (Figure 8).

## Interpretation

The results from the TeSS and TUQ surveys clearly demonstrate that both patients and telemedicine liaisons were highly satisfied with TeleMSK and its future use in providing consultations and care for patients. Participants within this study agreed that the use of telemedicine devices for consultations allowed improved access to healthcare services for patients and reduced time traveling to and from specialist appointments.

This reduction in travel time and improved efficiency in delivery of healthcare services will lead to a reduction in costs for patients and their families. Patients and families living in a rural or remote location may rely on private transportation companies for travel to and from consultation appointments, which on average costs $450 per appointment. Moreover, family members may need to take time off work to arrange transportation and accompany their loved one to consultation appointments. The use of TeleMSK markedly reduces these costs to patients and families by providing access to specialized health care remotely. In addition, often these patients attend their appointment either with a family member and/or a care attendant. There often is a barrier to obtain accurate care-related information from the patient and their companion. The TeleMSK eliminate this barrier by speaking directly to the frontline care providers. Information about the patient's recovery and future care instructions can be related directly to them.

The results of this study indicate that patients felt the quality of the interaction and care provided while using the TeleMSK technology was excellent. Patient-physician encounters did not appear to diminish while using the telecommunication system. The use of telemedicine in an orthopaedic setting was positive and quality of care was not compromised. Although many healthcare providers have concerns that patients prefer an in-person consultation, this study revealed that patients were very satisfied with an online encounter through TeleMSK.

The reports from telemedicine liaisons indicate the TeleMSK system is easy to use and learn as well as allowed for clear communication between the patient and physician. Satisfaction ratings from liaisons were high, and the system was generally found to be reliable. All telemedicine liaisons indicated that the TeleMSK visits were comparable with in-person consultations.

## Limitations

Owing to the small sample size of this study, which was conducted in a rural community, it is difficult to apply the results broadly. The results of patient and family satisfaction of TeleMSK may not be the same if applied to a large urban center. This study was conducted exclusively with patients living in long-term care. This population is unique because they typically have fewer financial resources, poorer overall health and mobility, and require family assistance to coordinate transportation to medical appointments. There was also only one orthopaedic surgeon involved, which may also skew the results.

## Conclusion

This study demonstrated high satisfaction rates among patients, family members, and telemedicine liaisons regrding the experience, comfort, and reliability in using a telemedicine program in an orthopaedic consultation setting. Telemedicine is a valuable platform for delivering orthopaedic consultations to patients living in long-term care. TeleMSK provides high-quality orthopaedic consultations that are comparable with in-person encounters. In fact, TeleMSK encounters for this patient population maybe better than in-person encounters. Often, the patients attended in-person appointments either with their family member or with an attendant. Owing to the patients' compromised mental function, and the fact that the family members and their attendants may not be fully involved in the patients' care, the information provided during the appointment may not be accurately relayed to care givers. In addition, instructions and expectations from the appointment may not be fully transferred to the frontline care providers when these patients are transferred back to the long-term care home. Some crucial information may be lost in this transfer. During the TeleMSK appointment, the frontline care providers are present. The questions and answers as well future plans are clearly communicated between the key stakeholders.

In addition to improving access to quality health care, TeleMSK has the potential to reduce the costs for the patient, patients' family members, and the healthcare system. It can also reduce the risks to the patient associated with transfers. The results from this study demonstrate the utility and efficiency of telemedicine for long-term care patients within an orthopaedic setting. With a focus on providing patient-centered care efficiently and effectively by using technology, there is a potential for further integration of TeleMSK in the general population.
